# Efficacy of Topical Folic Acid in the Healing of Deep Second-Degree Burn Wound via Redox Modulation in Rat

**DOI:** 10.1155/bmri/7337481

**Published:** 2025-07-20

**Authors:** Ali Dalir, Shabnam Pourmoslemi, Sara Soleimani Asl, Pari Tamri

**Affiliations:** ^1^Pharmaceutical Sciences Research Center, Institute of Cancer, Hamadan University of Medical Sciences, Hamadan, Iran; ^2^Department of Pharmacology and Toxicology, School of Pharmacy, Hamadan University of Medical Sciences, Hamadan, Iran; ^3^Department of Pharmaceutics, School of Pharmacy, Hamadan University of Medical Sciences, Hamadan, Iran; ^4^Department of Anatomical Sciences, School of Medicine, Hamadan University of Medical Sciences, Hamadan, Iran

**Keywords:** antioxidant, burn injury, folic acid, ROS, wound healing

## Abstract

Folic acid, synthetic form of folate, is necessary for synthesis and for repair of DNA. Studies have shown that folic acid enhances the DNA repair capacity of skin fibroblasts following tissue damage. The research objective was to assess the efficacy of topical folic acid on the treatment of second-degree burn wound in a rat model. The second-degree burn wounds were induced on the dorsal skin of Wistar rats. Animals were randomly placed into five groups (*n* = 5) as follows: (1) non-treatment group, (2) cream base treated group, (3) silver sulfadiazine (SSD) 1% treated group, (4) folic acid 1% cream treated group, and (5) folic acid 4% cream treated group. The healing effects of folic acid were assessed by monitoring the wound contraction rate, measuring the tissue content of hydroxyproline, and conducting a microscopical study of wound healing in experimental groups. For evaluation of antioxidant properties of folic acid, the total antioxidant capacity (TAC) and the amount of reactive oxygen species (ROS) in tissue samples were measured. Our results revealed that topical folic acid 1% and 4% (w/w) significantly (*p* < 0.05) accelerated wound contraction and re-epithelialization rates, enhanced hydroxyproline content of tissue, and decreased the median time to complete wound closure compared to non-treatment and cream base treated groups. Furthermore, 1% and 4% folic acid creams significantly (*p* < 0.05) increased the TAC content of skin tissue and suppressed the ROS levels compared to non-treatment and cream base groups. In conclusion, folic acid is capable of accelerating the burn wound healing process possibly via modulating oxidative stress.

## 1. Introduction

Burn is a tissue damage that results from contacting heat or exposure to severe cold, electrical current, or radiation. Thermal injuries are the most common form of burns [1]. Burns are among the primary causes of severe trauma and long-term physical disability worldwide [2]. Burn severity is assessed by determining the burn degree. Burns are classified into four degrees based on burn depth [3]. First-degree burn is one in which only the epidermis layer is damaged; second-degree burns are those in which the epidermis and some parts of the dermis are affected by thermal injury; third-degree burns involve injury to the epidermis and dermis, along with complete destruction of sensory nerves; and fourth-degree burn is one in which the skin, fascia, muscle, and even bone are affected by thermal injury [4].

Partial thickness (second-degree) burns are the most frequently occurring type of burn injuries. These burns are classified based on depth and severity as superficial partial thickness burns and deep partial thickness burns. In superficial partial thickness burns, thermal injury affects the epidermis and papillary layer of the dermis. They can also be severe and even life threatening when the epidermis and reticular layer of dermis are damaged by thermal injury; in this case, they are called deep second-degree burns [5]. Both minor and major burns (greater than 15% total body surface area) initiate the wound healing process, which includes continuous and intersecting phases of inflammatory response, proliferative or granulation, and maturation [4]. By using safe and effective treatment methods, wound healing can be accelerated. The most commonly used dressing and topical agents for burn treatment include silver-containing agents, petroleum gauze, and mafenide. Despite having beneficial effects, they also have disadvantages. Silver sulfadiazine (SSD) is an FDA-approved drug for the prevention and treatment of second- and third-degree burn wound infections. SSD is the most commonly used topical treatment for burns; however, it is associated with side effects and toxicity. SSD slows epithelialization; in which case, it may be necessary to stop it. In addition, its frequent use causes a pseudoeschar formation over the burn site. Also, in the case of systemic absorption, it leads to hematological and dermatological complications [6, 7]. Therefore, the efforts to find new effective, safe, and inexpensive treatments for burns are very important.

Folic acid is a water-soluble vitamin that belongs to the B complex family of vitamins. Folic acid (vitamin B9) is involved in the production of purine and pyrimidine; therefore, it plays a crucial role in DNA synthesis and proper cellular functions. Folic acid scavenges free radicals by providing methyl donors, including methionine and S-adenosyl methionine (SAM) [8, 9], and in fact, it is a strong scavenger of free radicals. It has been demonstrated that folic acid has antiaging and wound healing properties [10]. The results of several studies indicated that folic acid supplementation improved the oxido-redox status of the body by increasing antioxidant markers such as glutathione (GSH) and by decreasing oxidant markers such as malondialdehyde (MDA) [8]. In addition, it has been revealed that folic acid deficiency significantly impairs the synthesis of collagen, elastin, and noncollagen dermal proteins [9].

Previous studies have examined the effects of oral folic acid on impaired wound healing in diabetic mice [11], as well as the effects of high doses of folic acid supplementation on the early stages of diabetic foot ulcers [12]. In addition, Duman et al. evaluated the effects of topical folinic acid (a 5-formyl derivative of tetrahydrofolic acid) on wound healing in rats. The results of the above studies confirmed the wound healing effects of folic acid in animal models and in humans, but no study has been conducted to date to investigate the effects of topical folic acid on burn wound healing. According to the results of previous studies and considering the antioxidant properties of folic acid as well as its role in protein synthesis, in particular collagen, in this experimental research, we assessed the efficacy of folic acid on the treatment of burns in an animal model of second-degree burn.

## 2. Materials and Methods

### 2.1. Animals

Twenty-five male and female Wistar rats (8–12 weeks, weighing range of 180–220 g) were acquired from the Animal Care and Reproduction Center of Hamadan University of Medical Sciences. Ten days before the start of the study, the animals were transferred to individual cages with standard temperature (22 ± 2°C) and humidity (55% ± 5) conditions and were provided with sufficient food and water. The animal use protocol was approved by the Animals Ethics Committee of Hamadan University of Medical Sciences (ethical code: IR.UMSHA.REC.1401.757).

### 2.2. Preparation of Folic Acid Cream

Folic acid 1% and 4% creams were prepared by dispersing 1 and 4 g of folic acid fine powder (Sigma-Aldrich, purity ≥ 97%, CAS Number: 59-30-3) in 99 and 96 g of cream base, respectively. The stability of the creams was investigated at different temperatures (4°C, 25°C, and 35°C, respectively) for 5 weeks. The physical stability of the folic acid creams was assessed by visual observation in normal room light. The observations showed that the prepared creams were physically stable at different temperatures and that the appearance of the creams did not change during the experiment. Microbial control was performed to evaluate the existence of *Staphylococcus aureus*, *Pseudomonas aeruginosa*, *Salmonella*, and *Escherichia coli* in folic acid and base creams. No growth of bacteria was observed.

### 2.3. Burn Wound Induction and Experimental Groups

Burn induction was performed after the animals were anesthetized with ketamine (90 mg/kg of 10% ketamine solution) and xylazine (10 mg/kg of 2% xylazine solution) injections. In summary, the hairs of the designated area for burn induction were removed, and the area was cleaned, and povidone iodine 10% was used for disinfecting the wound area. The burn injury was made by keeping a preheated round metal plate 1 cm in diameter in contact with animal skin for 15 s. Previously, the metal plate was heated in boiling water until its temperature reached 100°C [13, 14]. Two burn injuries were created on the dorsal skin of each rat. Immediately after the burn induction, analgesia with an intramuscular injection of piroxicam (1 mg/kg) was carried out and was continued for 3 days in a row. Twenty-five animals were placed into five groups as follows: nontreatment group, cream base group, SSD 1% cream group (positive control), and folic acid 1% and 4% groups (test groups). Treatments were applied topically twice a day. There were five animals in each group.

### 2.4. Wound Contraction Rate Monitoring

Wound contraction is a critical phase of the healing process, reducing wound size by drawing the edges toward the center and facilitating faster closure [15]. Wound contraction rates were monitored by photographing the wounds every 3 days. The area of each wound was calculated by ImageJ software, and the rate of wound contraction was calculated using the following formula [1]:

 $$\begin{array}{c} \text{Wound contraction rate}=\frac{\left(\text{wound surface area at first day}-\text{wound surface area at specific day}\right)}{\text{wound surface area at first day }}\times 100. \end{array}$$

### 2.5. Histopathological Assessment

Microscopic studies of wound healing process were performed on samples collected on Day 7 after burn injury and also on samples obtained after complete wound closure. The collected specimens were stained using hematoxylin and eosin (H&E) and also using Masson's trichrome method. The histopathological study included inflammation, angiogenesis, collagen synthesis and deposition, and epithelialization [16].

### 2.6. Measurement of Reactive Oxygen Species (ROS) Production in Tissue Samples

ROS levels of skin tissue specimens were determined by using dichlorodihydrofluorescein diacetate (DCFH-DA) [17]. Briefly, the fresh skin samples were ground into small pieces using a mortar, then homogenized in extraction buffer (containing 10 mM Tris, pH 7.4, 150 mM NaCl, and 1% Triton X-100) using a glass homogenizer. The homogenous tissue mixture was centrifuged for 20 min at 1000×g, and the supernatant fluid was collected. Twenty-five microliters of supernatant fluid was added to a mixture of 5 *μ*L of 2⁣′,7⁣′-dichlorodihydrofluorescein (DCFH) and 100 *μ*L of assay buffer. After 15 min incubation of solution in dark, the absorbance of samples was measured using a microplate reader at an excitation wavelength of 485 nm and an emission wavelength of 530 nm.

### 2.7. Measurement of Hydroxyproline Content of Tissue Specimens

Biochemical measurement of hydroxyproline is one of the reliable and affordable methods to determine collagen quantity. For the measurement of hydroxyproline concentrations in tissue samples, Kiazist Hydroxyproline Assay Kit (Kiazist Life Sciences, Iran) was used, and the measurement was performed pursuant to manufacturer's instructions. Briefly, tissue specimens were hydrolyzed in HCL to release hydroxyproline; then, hydroxyproline was converted to pyrrole by reacting with chloramine T, which reacted with Ehrlich's reagent and formed chromophore that was detected at 540 nm. Hydroxyproline concentrations were determined from standard curve.

### 2.8. Total Antioxidant Capacity (TAC) Assay

TAC of tissue samples was determined using the ferric reducing antioxidant power (FRAP) assay, as described previously [18]. Briefly, tissue samples were homogenized in a homogenization buffer (20 mM Tris and pH 7.8, with protease and phosphatase inhibitors) and centrifuged to obtain the supernatant. For the preparation of FRAP reagent, 10 parts of 300 mM acetate buffer (pH 3.6), 1 part of 10 mM 2,4,6-tripyridyl-s-triazine (TPTZ) in 40 mM HCl, and 1 part of 20 mM ferric chloride (FeCl_3_) were mixed together. The supernatant (50 *μ*L) was mixed with the FRAP reagent (950 *μ*L), and after 30 min of incubation at 37°C, the absorbance of samples was read at 593 nm using a spectrophotometer, and the antioxidant capacity was reported as micromole of Fe^2+^ equivalents per gram of tissue.

### 2.9. Statistical Analysis

Data analysis was carried out using statistical software of GraphPad 6 and SPSS 21. One-way ANOVA method followed by multiple comparison test was used to analyze the collected data. Data were reported as mean ± SEM. A *p* value less than 0.05 indicated the statistically significant difference between groups.

## 3. Results

### 3.1. Wound Contraction Rate

**Figure 1:**
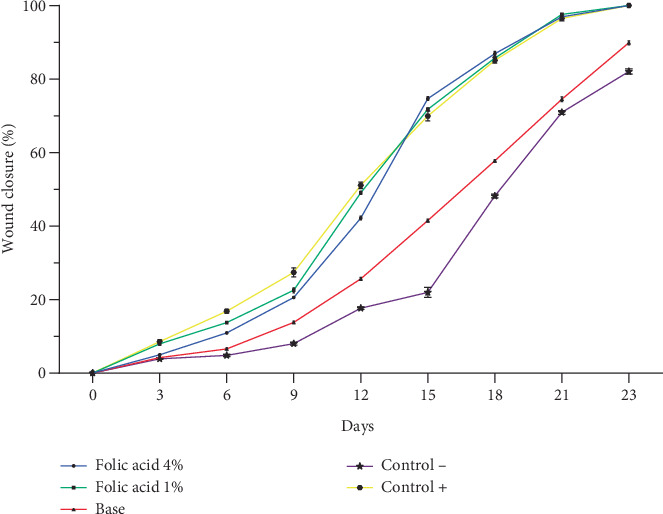
Wound contraction rates of experimental groups from day 3 to day 23 after burn induction. Data is represented as mean ± SEM. Control−, nontreatment group. Control+, SSD-treated group.

Assessment of wound contraction rate in experimental groups revealed that folic acid 1% and 4% creams enhanced the wound contraction rate. A significant increase (*p* < 0.05) in wound contraction rate was observed in folic acid–treated groups, compared with nontreatment and cream base–treated groups from Day 6 postinjury. However, there were no significant differences in the rate of wound contraction among folic acid 1%, folic acid 4%, and SSD-treated groups (Figure 1).

**Figure 2:**
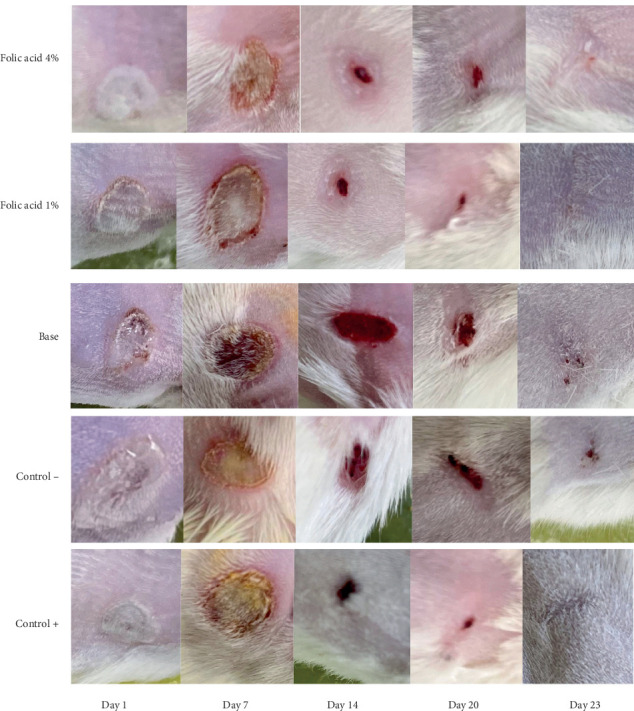
Images of wounds of burn tissue in different experimental groups on Days 1, 7, 14, 20, and 23 after burn injury. Control−, nontreatment group. Control+, SSD-treated group.

The median time to complete wound closure in studied groups was as follows: folic acid 1% (21.05 ± 1.04 days), folic acid 4% (21.83 ± 0.98 days), SSD (21.5 ± 1.0 days), cream base (26.33 ± 1.96 days), and nontreatment (26.83 ± 1 days) .The durations of complete wound closure in the animals treated with folic acid 1% and 4% and with SSD were significantly shorter than those of animals that received no treatment or were treated with cream base. Increasing the concentration of folic acid did not enhance its efficiency, and in fact, there was no significant difference in average time of complete wound closure between folic acid 1% and 4% treated animals. Images of the wounds of the studied groups on different days are shown in Figure 2.

### 3.2. Histopathological Findings

**Figure 3:**
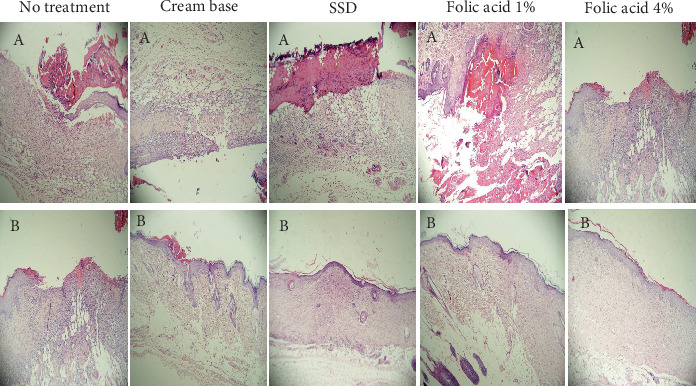
Micrographs of hematoxylin and eosin (H&E)–stained burned tissue specimens of experimental groups on (A) Day 7 and (B) after complete wound closure.

**Figure 4:**
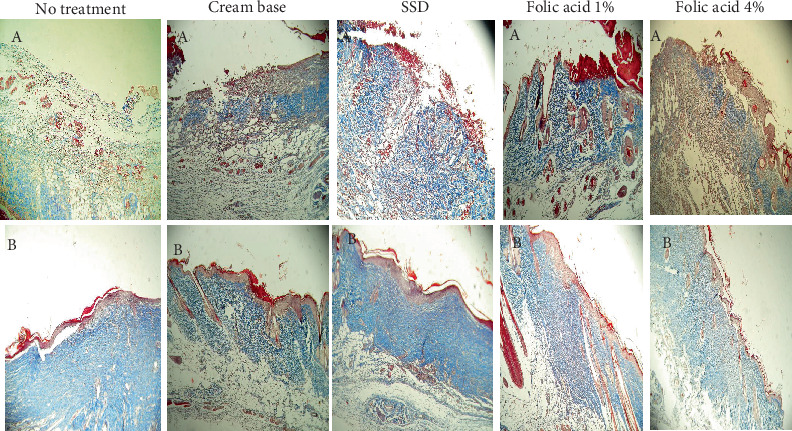
Micrographs of Masson's trichrome–stained burned tissue specimens of experimental groups on (A) Day 7 and (B) after complete wound closure.

Histopathological status of tissue samples was evaluated after staining with H&E and Masson's trichrome staining methods (Figures 3 and 4). Histopathological study of specimens on 7th day postinjury revealed enhanced epithelialization and angiogenesis in folic acid 1% and 4% treated rats, compared with nontreatment and cream base–treated groups. In addition, the amount of collagen deposition in the nontreatment group was lower than that of the other groups.

Evaluation of histopathological status of tissue samples after complete wound closure indicated that the thickness of the epidermis and the amount of collagen accumulation in samples obtained from animals treated with folic acid 1% and 4% were more than those of the other groups.

### 3.3. ROS Levels

**Figure 5:**
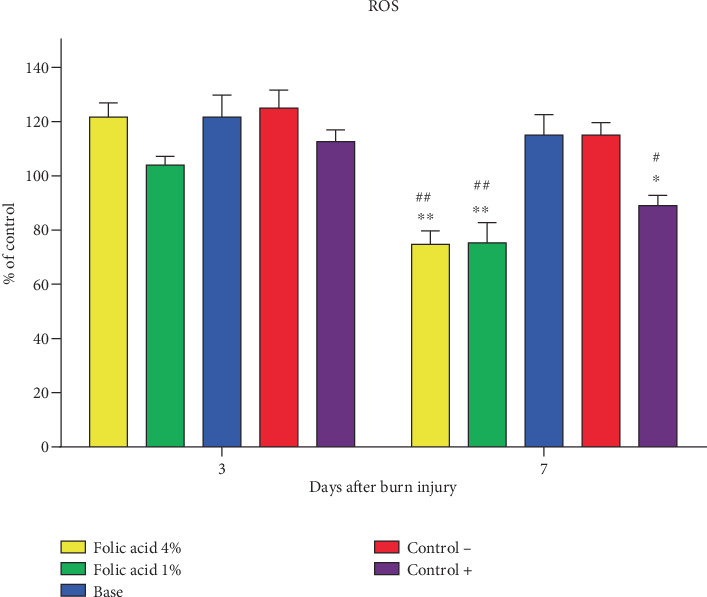
Comparison of ROS levels of tissue specimens of experimental. Data expressed as mean ± SEM. ⁣^∗^*p* < 0.05 and ⁣^∗∗^*p* < 0.01 represent significant difference from the nontreatment group; #*p* < 0.05 and ##*p* < 0.01 represent statistical significant difference from the cream base group. Control−, nontreatment group. Control+, SSD-treated group.

Measurement of ROS levels in tissue specimens on the 3rd day postburn wounding revealed that there were no significant differences in ROS levels among the different groups. On the 7th day postburn injury, ROS levels in tissue samples obtained from folic acid 1% and 4% treated animals were significantly (*p* < 0.01) suppressed compared to nontreatment and cream base–treated groups. ROS levels of tissue samples treated with SSD were also significantly (*p* < 0.05) reduced compared to nontreatment and cream base–treated groups. However, no significant differences were observed among folic acid 1%, folic acid 4%, and SSD-treated groups regarding ROS levels (Figure 5).

### 3.4. Hydroxyproline Concentration Assay

**Figure 6:**
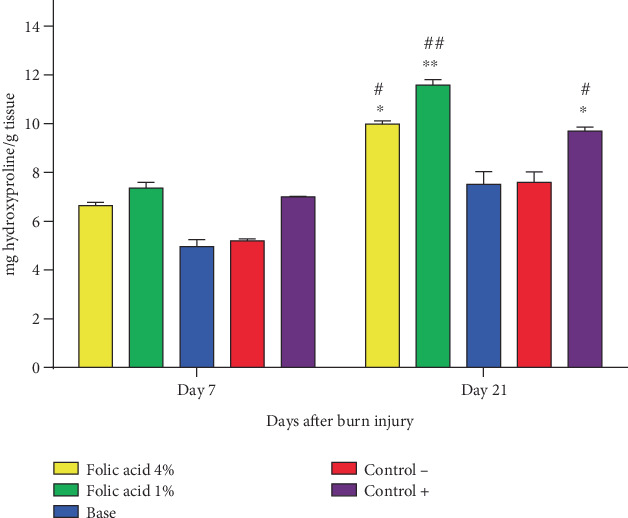
Hydroxyproline concentrations in tissue samples collected from studied groups. Data expressed as mean ± SEM. ⁣^∗^*p* < 0.05 and ⁣^∗∗^*p* < 0.01 represent statistical significant difference from the nontreatment group; #*p* < 0.05 and ##*p* < 0.01 represent statistical significant difference from the cream base group. Control−, nontreatment group. Control+, SSD-treated group.

The hydroxyproline concentrations at skin tissue specimens obtained from the studied groups were measured on 7th and 21st days after the burn induction, and the results are shown in Figure 6. There was no significant difference in terms of tissue hydroxyproline content among different groups on Day 7, while on Day 21, the hydroxyproline concentrations significantly increased at tissue specimens of folic acid 1% (*p* < 0.01), folic acid 4% (*p* < 0.05), and SSD (*p* < 0.05)-treated groups compared to nontreatment and cream base–treated groups.

### 3.5. TAC Assay

**Figure 7:**
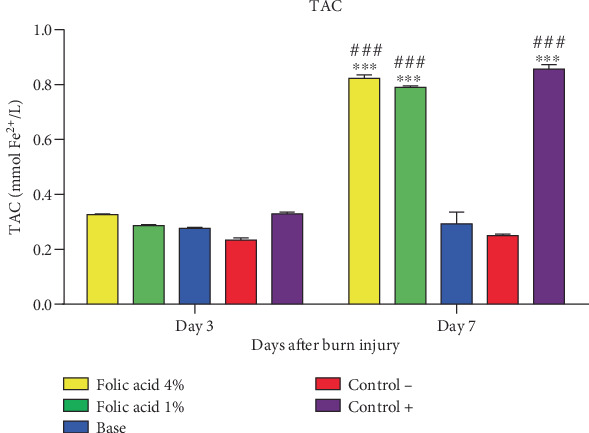
Total antioxidant capacity (TAC) of tissue specimens of experimental groups. ⁣^∗∗∗^*p* < 0.001 represents statistical significant difference from the nontreatment group, and ###*p* < 0.001 represents statistical significant difference from the cream base group. Control−, nontreatment group. Control+, SSD-treated group.

TAC concentrations of tissue samples treated with folic acid 1% and 4% and with SSD were significantly (*p* < 0.01) elevated, compared with those of nontreatment and cream base–treated groups on 7th day postinjury. TAC levels of skin tissue were not significantly affected by folic acid or SSD on the 3rd day after burn induction (Figure 7).

## 4. Discussion

Burn injuries are one of the most frequently occurred forms of injury that can lead to serious damage to body organs [3]. Healing a burn is a complex prolonged process that involves repair and restoration of lost tissue [19]. The depth of the burn is the most important factor for the classification of burns, and based on the depth of the burns, they are divided into first- to fourth-degree burns. Second-degree burns are the most frequently seen type of burn in a clinical setting, and they are hard to manage [20]. The wound healing rate of second-degree burn is slow, and improving the healing process is the focus of research in the field of burns [21]. The improvement of the wound healing process by folic acid has been reported in previous studies [11, 12], but so far, the effects of topical folic acid on burn wound healing have not been investigated.

The present experimental study was conducted to assess the effects of topical folic acid on deep second-degree burn in rats. The effects of folic acid were investigated by monitoring wound closure and re-epithelialization rates, hydroxyproline content of tissue, and ROS production in burned tissue. In addition, the TAC of burned tissue exposed to topical folic acid was assessed compared to control groups (nontreatment, cream base, and SSD-treated groups).

In this study, we used SSD as a positive control. SSD 1% cream has been the most widely used topical antimicrobial agent for infection control in burn patients. However, recently, it has been reported that SSD has considerable disadvantages such as the formation of a pseudoeschar layer on the burn area and delayed wound healing [22, 23]. Nevertheless, based on the results of this research, SSD effectively promoted burn wound healing, albeit with minor complications including delayed re-epithelialization, a thinner epidermal layer, and less organized collagen deposition compared to folic acid treated groups. These findings underscore the potential advantages of folic acid in promoting more robust tissue regeneration and superior wound quality compared to SSD.

Folic acid 1% and 4% stimulated the wound contraction and re-epithelialization rate, which was confirmed by macroscopic and microscopical examinations. Epithelialization is the most essential part of wound healing which is used as a parameter to evaluate the success of wound healing. Without epithelialization, the wound cannot be considered repaired, and the openness of the wound provides the basis for wound infection and even systemic infection. Wound closure is a process that involves wound contraction and re-epithelialization. The mechanism by which folic acid increased the rate of wound contraction was not investigated in this study. Given that wound contraction requires migration of fibroblasts and keratinocytes, it seems that folic acid stimulated the migration of these cells to the wound site. However, further studies are needed to prove this.

Hydroxyproline contents of tissue samples obtained from folic acid 1% and 4% treated animals were significantly higher than those of nontreatment and cream base–treated groups on Day 21 after burn induction. Collagen is one of the main components of the extracellular matrix (ECM) and plays a crucial role in each phase of the wound healing process; it involves inflammation and granulation tissue formation and provides tensile strength during tissue remodeling [24]. Hydroxyproline measurement provides a reliable and accurate estimate of the collagen content of tissue samples, and it is a widely used method to quantify collagen [25]. A higher concentration of hydroxyproline has a direct relationship with increasing the wound closure rate, as the findings of this study showed that the wound closure rate increased in groups with a higher tissue collagen content. The observed increase in collagen levels in response to folic acid treatment may be attributed to its pivotal role in one-carbon metabolism, which is crucial for DNA synthesis, repair, and methylation. Folic acid contributes to the production of SAM, a universal methyl donor involved in the epigenetic regulation of gene expression, including genes encoding collagen, such as COL1A1 and COL3A [26]. Additionally, folic acid supports the biosynthesis of amino acids, particularly proline and glycine, which are essential components of the collagen triple helix [27]. Moreover, folic acid may indirectly modulate the TGF-*β*/Smad signaling pathway, a key regulator of collagen deposition during wound healing; nevertheless, further studies are needed to prove this.

Development of oxidative stress and production of ROS play a critical role in the orchestration of wound healing and repair. ROS are involved in inflammation and angiogenesis regulation and effective wound healing [28]. This study demonstrated that folic acid reduced ROS levels in burn tissue 7 days after burn induction, suggesting its beneficial role in burn wound healing. Elevated ROS in burn injuries contribute to oxidative stress, which can delay healing by causing cellular damage and prolonging inflammation [29]. The antioxidant action of folic acid likely mitigates this effect, fostering a more favorable environment for tissue repair. Additionally, folic acid aids in essential healing processes such as cell proliferation, angiogenesis, and collagen synthesis [30]. These findings indicate that folic acid may be a valuable addition to burn care, promoting recovery by reducing oxidative stress. The antioxidant properties of folic acid and its important role in proper cellular function have been confirmed in various studies; for example, Zhao et al. evaluated the effects of folic acid in the regulation of mitophagy, in vivo and in vitro, and they concluded that folic acid improved mitophagy remodeling by scavenging ROS and promoting hepatic homocysteine metabolism [31]. The findings of another study showed that folic acid is an essential nutrient to combat ROS insults and it regulates redox, GSH biosynthesis and transporting system, and process of mitochondrial GSH recycling [32]. Our findings also revealed that folic acid enhanced TAC of the burn tissue. Therefore, it can be concluded that folic acid has improved the burn wound healing process by creating a balance between oxidative stress and the antioxidant system.

## 5. Conclusion

Topical folic acid 1% and 4% accelerated epithelialization and wound closure rates, enhanced collagen deposition, and modulated balance between oxidation stress and antioxidant system. The effectiveness of folic acid cream and topical SSD on burn wound healing was comparable; however, folic acid has advantages over SSD, such as a better safety profile and noninterference in the wound healing process. It can be concluded that folic acid has a good potential to accelerate burn wound healing and it is eligible to be considered a candidate for the preparation of appropriate topical formulations for the effective treatment of burns and wounds.

## Data Availability

The data that support the findings of this study are available on request from the corresponding author. The data are not publicly available due to privacy or ethical restrictions.
